# Within-sample variability of steroid and thyroid metabolite measurements in faeces of Northeast Pacific resident killer whales (*Orcinus orca*)

**DOI:** 10.1093/conphys/coaf070

**Published:** 2025-10-09

**Authors:** Kaitlin E Yehle, Valentina Melica, Colin J Brauner, Sheila J Thornton

**Affiliations:** Department of Zoology, 6270 University Blvd, University of British Columbia, Vancouver,V6T 1Z4 Canada; Fisheries and Oceans Canada, 4160 Marine Drive, West Vancouver, British Columbia, V7V 1H2 Canada; Fisheries and Oceans Canada, 4160 Marine Drive, West Vancouver, British Columbia, V7V 1H2 Canada; Department of Zoology, 6270 University Blvd, University of British Columbia, Vancouver,V6T 1Z4 Canada; Fisheries and Oceans Canada, 4160 Marine Drive, West Vancouver, British Columbia, V7V 1H2 Canada

**Keywords:** Faecal corticosterone, faecal hormones, faecal thyroid metabolites, killer whale, *Orcinus orca*

## Abstract

Faecal hormone metabolite (FHM) analyses are increasingly used as a non-invasive method to evaluate physiological stress in wild populations, especially those of conservation concern. In cetaceans, faecal collection from the ocean surface results in considerable variation in sample volume and density. Knowledge of the distribution of hormone metabolites within a faecal sample is limited, but is an important consideration when interpreting values. Here we investigated the variability of glucocorticoid (fGCM) and thyroid (fTHM) metabolites within fish-eating resident killer whale faeces by comparing mean concentration, standard deviation (SD) and coefficient of variation (CV) among three treatment groups: sub-samples, pooled sub-samples and homogenized pooled sub-samples from the same defecation event. No significant difference was found in the mean concentration of fGCM and fTHM across treatment groups. The mean SD for fGCM was significantly higher in sub-samples than in pooled and homogenized treatment groups (*P* < 0.05), while differences in the mean SD of fTHM were not significant among treatment groups. Overall, the CV of FHM measurements was reduced to less than 15% and 10%, respectively, by pooling and homogenizing the sub-samples prior to analysis. We found high correlation in fGCM and fTHM across all treatments, suggesting that values from sub-samples were generally representative of the overall faecal sample. These findings help guide methods for processing cetacean faecal samples and interpreting associated FHM data.

## Introduction

Faecal hormone metabolite (FHM) analyses are commonly used as a non-invasive method to assess stress and reproductive physiology in domestic and wild, free-ranging animals (Möstl and Palme, 2002; Goymann, 2012; Palme, 2019). Compared to hormone measurements from traditional matrices such as serum or plasma, measurements from faeces offer a less-invasive and integrated measure of endocrine activity over a longer time frame (that of gut transit time), which is particularly useful for investigating chronic stress in large-bodied mammals (Möstl and Palme, 2002; Palme, 2019; Steinman *et al.,* 2020). The negative health implications of chronic stress are of concern for at-risk marine mammal populations (Fair and Becker, 2000; Wright *et al.,* 2011), and FHM analysis is increasingly used as a means of assessing anthropogenic stress in threatened and endangered cetacean populations (Rolland *et al.,* 2017; Wasser *et al.,* 2017).

While FHM measurements have great potential for assessing stress in wild cetaceans (Rolland *et al.,* 2012; Wasser *et al.,* 2017; Lemos *et al.,* 2020), collecting faeces in a marine environment presents its own unique challenges. For instance, detection and retrieval of faecal material remains opportunistic and can be significantly hindered by unfavourable environmental conditions (e.g. weather, swell, sea state). Even with the use of techniques and instruments—such as scent-detection dogs (Ayres *et al.,* 2012) and unmanned aerial vehicles (UAVs; Torres *et al.,* 2018; Baird *et al.,* 2022)—to facilitate quick detection and reduce disturbance to the animals, faecal plumes tend to disperse and rapidly sink, often leading to the recovery of small volumes (Melica and Thornton, 2024). This contrasts with FHM studies in terrestrial animals, in which the entire faecal deposit is often collected, homogenized and analyzed (Wasser *et al.,* 2000; Millspaugh and Washburn, 2003).

The distribution of hormone metabolites within faeces is not well studied. Despite acknowledgement that larger faecal samples may provide more representative FHM values, (Ayres *et al.,* 2012; Hunt *et al.,* 2019; Lemos *et al.,* 2020), no studies have directly examined the distribution of hormone metabolites within cetacean faeces. Studies in terrestrial mammals have found unequal distribution of oestrogen and progestogen (Brown *et al.,* 1994; Wasser *et al.,* 1996) and glucocorticoid (Millspaugh and Washburn, 2003; Parnell *et al.,* 2015) metabolites in faecal pellets of several species, and recommend measuring FHMs in well mixed, dried faecal powder from pre-mixed wet samples for highest accuracy (Wasser *et al.,* 1996; Millspaugh and Washburn, 2003). The uneven distribution of hormone metabolites in cetacean faeces (e.g. hormone “hot spots”) may lead to highly variable measurements (Fernandez Ajó *et al.,* 2023) or add uncertainties around the determination of physiological state (Valenzuela-Molina *et al.,* 2018), particularly when only small, partial samples are collected. Therefore, understanding the distribution of hormone metabolites within faeces is an important consideration when sub-sampling for analysis.

**Figure 1:**
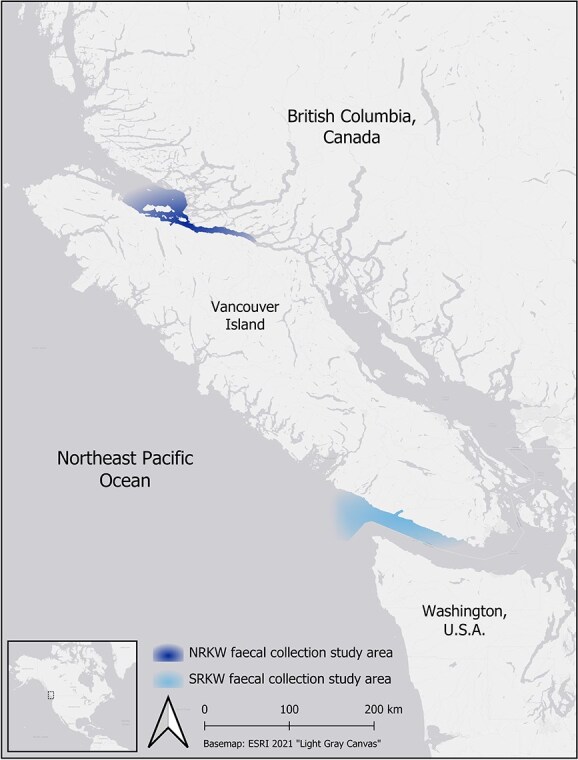
Study areas where faecal samples were collected from free-ranging Northern resident killer whale (NRKW; dark shading) and Southern resident killer whale (SRKW; light shading) populations in July, August and September 2018–2022.

Measures of faecal glucocorticoid metabolites (fGCM) and, more recently, faecal thyroid metabolites (fTHM) are of particular interest when investigating stress in aquatic mammals (Gobush *et al.,* 2014; Champagne *et al.,* 2018; Hunt *et al.,* 2019), as both glucocorticoids and thyroid hormones are involved in energy metabolism and fluctuate in response to environmental and other stressors (Kelly, 2000; Reeder and Kramer, 2005; Romero and Butler, 2007; Norris and Carr, 2013). For example, changes in concentrations of fGCM and fTHM have been observed in killer whales *(Orcinus orca)* in response to prey availability and vessel traffic (Ayres *et al.,* 2012). As thyroid function is related to energy intake and is sensitive to acute and chronic changes in quality and quantity of food (Dauncey *et al.,* 1983; Eales, 1988; Mullur *et al.,* 2014), fTHM is of interest particularly with respect to nutritional stress. However, compared to fGCM, fTHM are relatively less studied and their application to assess stress (specifically nutritional stress) is still exploratory in many cetacean species, with some studies reporting elevated thyroid metabolites associated with increased prey availability or body condition (Ayres *et al.,* 2012; Hunt *et al.,* 2019) and others reporting the opposite (Lemos *et al.,* 2020, 2021). The distribution of thyroid hormone metabolites within faeces may also differ from that of steroid metabolites and, to our knowledge, no studies to date have investigated the distribution of thyroid metabolites within faeces of any species, let alone faeces collected from water. A thorough understanding of FHM variability is necessary to ensure that collection and processing of faecal samples provides accurate and reliable measurements of FHMs.

In anticipation of applying FHM analysis to investigate stress in two free-ranging resident type killer whale populations, we investigated the “within-sample variability” of glucocorticoid and thyroid metabolites in killer whale faecal samples collected in the Northeast Pacific Ocean by comparing fGCM and fTHM concentrations in unmixed sub-samples to concentrations in pooled and homogenized treatments of the same faecal sample. In this paper, we set out to (i) examine and quantify the variability of glucocorticoid and thyroid metabolites within killer whale faeces and (ii) compare three methods for processing and aliquoting killer whale faecal samples for FHM analyses to determine which yields the most reliable measurements. We expected sub-samples to exhibit greater hormone variability compared to both pooled and homogenized treatments of the same faecal event, indicating that hormone metabolites are not evenly distributed in killer whale faeces and, therefore, that pooling and homogenizing sub-samples would be the preferred processing methods. However, we hypothesized that the natural variability of hormone metabolites in faeces would be relatively low compared to inter-sample variability, such that FHM values from small sub-samples would accurately reflect physiological activity.

## Materials and Methods

### Faecal sample collection

Faecal samples were collected from two fish-eating populations of free-ranging killer whales in the Northeast Pacific in the months of July, August and September from 2018 to 2022. Samples were collected from small vessels with twin outboard engines, through a combination of opportunistic and targeted techniques (see Yehle, 2022). Southern Resident killer whale samples were collected off the southwest coast of Vancouver Island in Juan de Fuca Strait and surrounding waters, and Northern Resident killer whale sampling occurred off the northeast coast of Vancouver Island in Johnstone Strait and surrounding waters (Fig. 1). All faecal samples were collected under Fisheries and Oceans Canada Species at Risk Act (SARA) compliant licences XMMS 052018, XMMS 72019, XMMS 42021, XMMS 42022, and US NOAA/NMFS permit no. 21348, with Animal Use Protocol approvals from the DFO Pacific Region Animal Care Committee (AUP-18-003 and AUP-19-010).

Faecal sample collection, on-vessel processing and storage methods were adapted from Ayres *et al.* (2012) and Wasser *et al.* (2017). Samples were collected either at the surface (down to approximately 30 cm depth) using a wooden extension pole and mounting ring for a 1-L polypropylene tri-cornered beaker, or by skimming the beaker at the surface by hand. Seawater was carefully decanted from the beakers and faeces were poured into sterile 50-ml polypropylene centrifuge tubes. Sterile syringes were used to assist in the transfer as necessary. Samples were then centrifuged at sea at 1000 rpm for 5 min (12 V Universal Centrifuge, LW Scientific, GA, USA) and any extra seawater separated from the centrifugation process was decanted. This step served to remove as much seawater as possible before freezing for long-term storage, as the potential loss of hormone metabolites to surrounding seawater is not well studied (Hunt *et al.,* 2014). When centrifugation was deemed unnecessary (i.e. The sample was clearly separated from the seawater.), or when centrifugation was not possible or practical, sample tubes were allowed to settle for 5 min using the vibrations of the vessel before decanting excess seawater. A sterile cotton swab was then used to obtain a sub-sample for genetic analysis; DNA swabs were stored in dimethyl sulfoxide solution (20% DMSO in 5 M saturated saline solution) until extracted and genotyped for species, sex and individual identification.

To limit microbial metabolism at ambient temperature and its consequent action on stability of faecal hormones (Washburn and Millspaugh, 2002), samples were frozen to −80°C as quickly as possible, typically within one hour. Samples were frozen on board the vessel using dry ice or in a dry shipper and then transferred to a −80°C freezer (Stirling Ultracold ULT25) at the end of each day.

### Sample processing & experimental design

Thirteen samples were selected for the study, according to the following criteria: (i) total volume greater than 30 ml wet faeces and (ii) sample was stored in three or more sub-sample tubes. Genetic results confirmed the thirteen samples included faeces from ten individuals, including a mix of both females and males, and five individuals from each population (Table 1).

**Table 1:** Metadata for thirteen killer whale (*Orcinus orca*) faecal samples from 10 individuals used to investigate within-sample variability of faecal glucocorticoid and thyroid metabolites; including a unique killer whale ID (assigned alphabetically in chronological order of sample collection), the population (Southern Resident killer whale; SRKW, Northern Resident killer whale; NRKW), sex (female; F, male; M or unknown) and the number of samples from each individual

Killer whale alphabetical ID	Population	Sex (F/M)	Samples (*n*)
A	SRKW	F	1
B	SRKW	M	2
C	NRKW	F	1
D	NRKW	F	2
E	SRKW	Unknown	1
F	NRKW	F	2
G	NRKW	Unknown	1
H	NRKW	M	1
I	SRKW	M	1
J	SRKW	F	1

**Figure 2:**
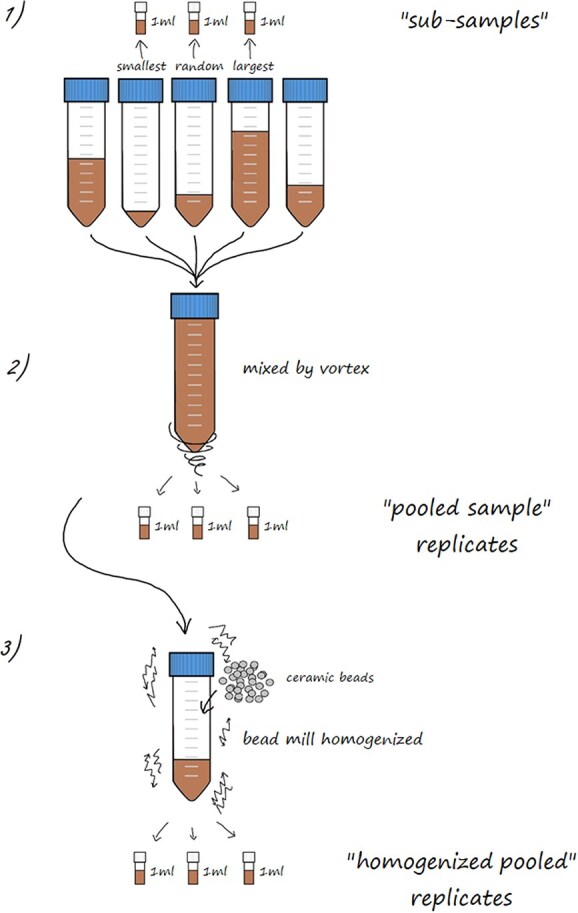
Faecal sample processing diagram depicting the methodology used to assess within-sample variation of faecal hormone metabolites for a single faecal sample: 1) three 1-ml sub-samples were made from the smallest, largest and a third random sub-sample tube; 2) three 1-ml pooled sample replicates were made from the remaining faeces from all sub-sample tubes combined together in a single tube and mixed by vortex; 3) if applicable (*n* = 7), three 1-ml homogenized pooled replicates were made from a portion of the pooled sample, which was then bead mill homogenized.

Sub-sample tubes were thawed in a cool water bath. Thaw time ranged from 10 to 145 min depending on sample tube volumes. Sub-sample tubes were then centrifuged at 2500 rpm for 20 min (Megafuge 2.0 R, Baxter Scientific) and any additional seawater separated was decanted off to minimize inflation of the apparent dried faecal mass due to salt content (Ayres *et al.,* 2012; Wasser *et al.,* 2017; Hunt *et al.,* 2019). Each sub-sample tube was briefly mixed by vortex. Then, a sterile syringe (1 ml) was used to aliquot 1 ml of faeces from each of three of the sub-sample tubes into pre-weighed 2.0-ml polypropylene cryogenic vials. The three sub-sample tubes selected to evaluate sample variation were: (i) the tube with the largest volume of stored faeces, (ii) the tube with the smallest volume of stored faeces, and (iii) a third, randomly selected tube from the remaining options. The smallest and largest volume tubes were chosen to capture the hypothesized variation of volume that may occur during faecal sample collection. Once these 1 ml “sub-samples” were removed, the remaining faecal contents from all the sub-sample tubes were pooled together, vortexed, and three 1 ml “pooled sample” replicates from the whole, mixed sample were partitioned into 2.0-ml vials using a sterile syringe. Any hard prey fragments, fish bones or parasites found during sample processing were removed. To evaluate the effect of an additional homogenization step, seven samples were then homogenized (Bead Ruptor 96, Omni International). A 15–25 ml volume of faeces was aliquoted to a new 50-ml centrifuge tube containing 70 ceramic beads (2.8 mm) and homogenized at 1500 rpm for 30 s (OMNI™ International 98 well bead ruptor). Three 1 ml replicates from this “homogenized pooled” sample were partitioned using a sterile syringe. The three treatments are hereafter referred to as: (i) sub-samples, (ii) pooled sample replicates, and (iii) homogenized pooled replicates (Fig. 2). All aliquots were weighed and re-frozen at −80°C until further analysis and all subsequent processing (hormone metabolite extraction and assay) was identical for each aliquot.

### Hormone metabolite extraction

Preparation of faecal aliquots for hormone extraction was performed according to methodology in Wasser *et al.* (2017). Specifically, faecal aliquots were removed from −80°C and immediately lyophilized at −48°C under vacuum seal (Labconco FreeZone 12) for 48 h or until completely dry (Wasser *et al.,* 1993, 2000). Preliminary tests of 1–2 ml aliquots lyophilized and weighed after 24, 48 and 72 h indicated no sample mass changes after 48 h. Lyophilized aliquots were brought to room temperature under desiccation, then were pulverized using 2–5 zirconia beads (2.0 mm) in a bead ruptor (SPEX Geno Grinder or an OMNI 98 International) at 1500 rpm for 45 s. For each aliquot, 0.02–0.11 g (mean ± SD: 0.07 ± 0.02 g) of dried faecal material was weighed and transferred into a new 50-ml polypropylene tube for extraction. To reduce sources of variability, attempts were made to ensure that the dry mass of all treatment aliquots for a given sample were matched as closely as possible (typically within 0.02 g).

The method used for extraction of hormone metabolites was adapted from Wasser *et al.* (2010)*.* Two extractions of each aliquot were performed using 15 ml of 70% ethanol solution (Molecular Biology Grade, Fisher Scientific #BP82031GAL). Tubes were sealed and vortexed at 2000 rpm for 30 min with 1 pulse/s on a Multi-pulse Vortexer (Glas-Col, Terre Haute, IN) (Wasser *et al.,* 2010). After pulse vortexing, tubes were centrifuged at 2500 rpm for 20 min. The supernatant was decanted into a 16 × 125 mm borosilicate glass tube, and a second ethanol extraction was performed on the pellet following the above methodology. Equal volumes of the first and second extracts were combined and vortexed, creating final extracts at 1:30 dilution, which were stored at −20°C until the evaporation step. This two-step ethanol extraction procedure was used to ensure thorough extraction of thyroid metabolites, as thyroid hormone metabolites may still be detectable in the faecal pellet after a single extraction (Wasser, personal communication, 2020).

Extraction efficiency was measured twice using a pool of dried faeces (*n* = 13), using modified methods from Steinman and Robeck (2021). Each time, three replicates of ~80 mg were aliquoted from the pool and extracted as described above. A second set of three replicates from the pool (~80 mg) were spiked with 5 ng of corticosterone standard (Arbor Assays™) and 3 ng of T_3_ standard (Arbor Assays™) and were processed identically to the first set. Mean ± SEM recovery of corticosterone for spiked faecal samples was 108.3 ± 6.1% and of T_3_ was 92.1 ± 2.2%.

### Ethanol evaporation and resuspension

As ethanol may interfere with the enzyme immunoassays (EIA) used in this study, the volume of the [1:30] extract required for the desired assay dilution was transferred by pipette into a new borosilicate glass tube, dried down under nitrogen gas (20–360 min depending on volume; Labconco™ RapidVap™ Vertex™ Evaporator), sealed and stored at −20°C until assayed. On the date of assay, sample dried extracts were allowed to come to room temperature for 30 min, then were resuspended using the appropriate volume of assay buffer (Arbor Assays™ cat. # X065) to obtain the desired dilution. Reconstituted extracts were briefly vortexed individually, followed by 20 min of sonication (ultrasonic cleaner, VWR), then 20 min of pulse vortexing (2000 rpm, 1 pulse/s), and were assayed within 2 h of reconstitution.

### EIA

All FHM measurements were expressed as *ng* hormone metabolites per *g* dried faeces. As measurements from faeces are most likely metabolites of the parent hormones, the terms used herein are faecal glucocorticoid metabolites (fGCM) and faecal thyroid metabolites (fTHM). fGCM and fTHM concentrations were measured using commercial EIA kits (respectively: #K014 corticosterone and #K056 triiodothyronine (T_3_), Arbor Assays™, Ann Arbor, MI, USA). Assays were performed following manufacturer protocols, with minor modifications. Standards were prepared using EIA kit provided hormone standards and used within 2 h of preparation. Microplates were shaken at room temperature for the EIA-specific time (1 or 2 h, depending on kit) at 500 rpm. TMB substrate was added using reverse pipetting technique to minimize the formation of bubbles. After the TMB substrate addition, plates were incubated for 30 min under a protective cover of darkness. The stop solution was then added, and plates were read for absorbance at 450 nm on a BioTek Synergy H1 microplate reader. FHM concentrations were calculated using the instrument software. All sample aliquots, standards, and controls were assayed in duplicate and any aliquot with a coefficient of variation (CV) of concentration between replicates >10% was repeated. All assay plates included a full standard curve, non-specific binding wells, total activity wells and blank wells. Samples were assayed between June 2021 and August 2024 and generally within one month of extraction; however, some samples were re-measured up to 14 months after extraction in order to measure all sub-samples, pooled sample replicates, and homogenized pooled replicates from the same faecal sample on a single assay plate—thus eliminating inter-assay CV as a source of variability in measurements.

#### Corticosterone

The Arbor Assays™ corticosterone EIA kit uses a polyclonal antibody to quantify total corticosterone in extracted faecal samples. This EIA kit has been previously validated with killer whale faeces (Steinman *et al.,* 2020) using a slightly different extraction protocol; therefore, analytical validations of parallelism and accuracy were performed (Grotjan and Keel, 1996; Palme, 2019). Serial dilutions of extract (1:1–1:256) from a pool of killer whale faeces (*n* = 13) demonstrated parallel displacement against the standard curve (*F-*test: *F*_1,7_ = 1.10, *P* = 0.90; Fig. S1). An accuracy test plotting the concentrations of a set of hormone standards spiked with equal volumes of the pooled extract near 50% binding (apparent dose) against a second set of standards spiked with equal volumes of assay buffer only (known dose) demonstrated linearity (linear regression: *y* = 1.08*x*—63.9, *r*^2^ = 0.995; Fig. S1). Further details on the analytical and biological validations for this assay can be found in Yehle (2022) and Melica and Thornton (2024). Mean intra-assay CV of concentration was 4.6% with up to 14.9% accepted for final values. The inter-assay CVs of concentration, based on biological controls made from pooled killer whale faeces extract, at approximately 40 and 60% antibody binding were 6.78 and 17.77%, respectively (*n* = 9). Final accepted values had antibody binding between 30 and 80%. Cross reactivity analyses of the corticosterone EIA were conducted by the manufacturer; values for non-corticosterone cross reactants range from 0.01 to 0.62% (Arbor Assay Catalogue # K014).

#### Triiodothyronine

The Arbor Assays™ T_3_ EIA kit uses a sheep antibody to T_3_ to measure total T_3_ in extracted faecal samples and has not been previously validated with killer whale faeces. Serial dilutions of extract (1:1–1:64) from a pool of killer whale faeces (*n* = 13) demonstrated parallel displacement against the standard curve (*F-*test: *F*_1,6_ = 0.87, *P* = 0.87; Fig. S1). An accuracy test plotting the concentrations of a set of hormone standards spiked with equal volumes of the pooled extract near 50% binding (apparent dose) against a second set of standards spiked with equal volumes of assay buffer only (known dose) demonstrated linearity (linear regression: *y* = 1.00*x*—79, *r*^2^ = 0.994; Fig. S1). Mean intra-assay CV of concentration was 6.1% with up to 16.7% accepted for final values. Inter-assay CVs of concentration, based on biological controls made from pooled killer whale faeces extract, at approximately 40 and 60% antibody binding were 17.87 and 23.81, respectively (*n* = 8). Final accepted values had antibody binding between 34 and 68%. Cross reactivity analyses of the triiodothyronine EIA were conducted by the manufacturer; cross reactant value for T_4_ was 0.88%, and for reverse T_3_ was <0.1% (Arbor Assay Catalogue # K056).

**Figure 3:**
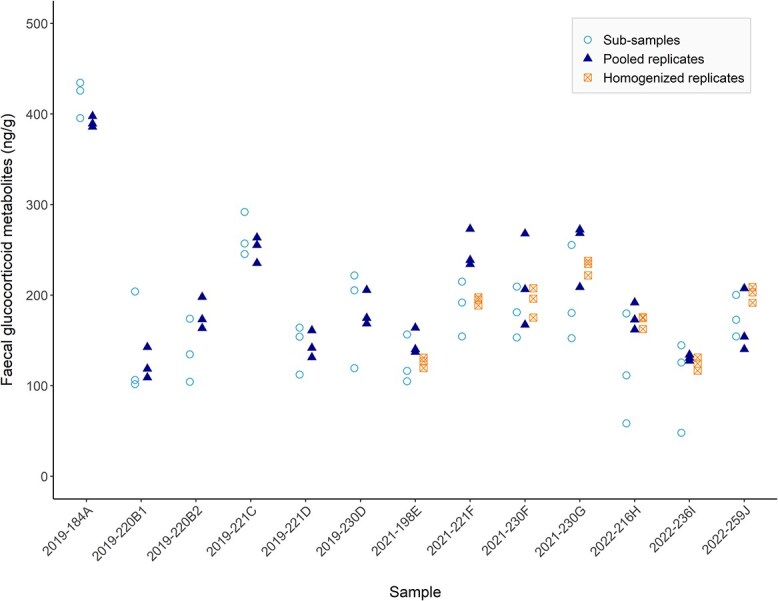
Concentration of faecal glucocorticoid metabolites (ng/g dried faeces) of 13 killer whale (*Orcinus orca*) faecal samples by treatment (sub-samples × 3, pooled sample replicates × 3, and homogenized pooled replicates × 3). Sample names indicate the year of collection followed by the ordinal date and a unique letter identification for each individual (data include a repeat sample from three individuals).

**Figure 4:**
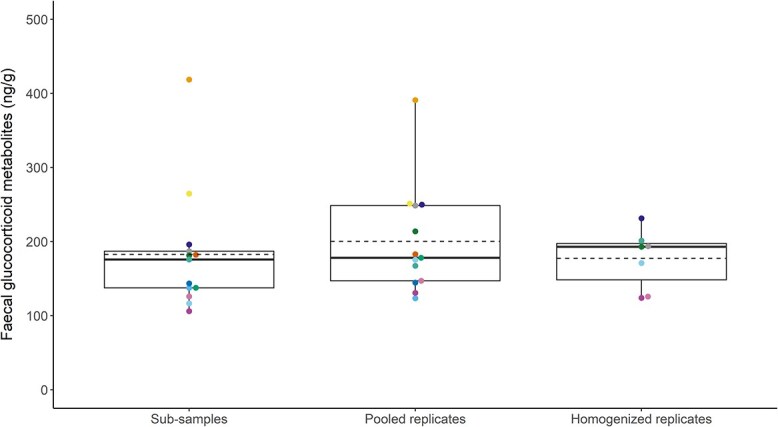
Concentrations of faecal glucocorticoid metabolites (ng/g) by treatment group. Model comparison indicated that treatment group was not a significant explanatory factor for mean concentration of fGCM (χ^2^(2) = 5.3, *P* = 0.07). Boxplots denote median (thick line), mean (dashed line), upper (75%) and lower (25%) quartile (boxes), and largest and smallest value within 1.5 times interquartile range below 25% and above 75% (whiskers). Boxplots are overlaid with each sample/treatment mean, with sample ID indicated by colour.

### Statistical analysis

Statistical analyses were performed in R (version 4.4.3; R Core Team, 2025) and approaches were adapted from Millspaugh and Washburn (2003). Mean concentrations were calculated for the three aliquots from each treatment, for each sample ID (Table S1). Differences in mean FHM concentrations across treatments were assessed using linear mixed effects models (R package: *lme4;*  Bates *et al.,* 2015), in which the killer whale ID, with nested sample ID, was considered as the random effect. For each hormone, the full model with treatment as predictor was compared to the null model using the Akaike’s information criterion with correction for small sample size (AICc) and the goodness of fit was assessed with a Chi-Squared (χ^2^) test. If significant, the full model was then analyzed with a post hoc test of Estimated Marginal Means (EMM) for pairwise comparison (R package:*emmeans:*  Lenth, 2024).

Variability was quantified using two parameters of spread: standard deviation (SD) and CV—the percentage ratio of SD to the mean). SD and CV were calculated for the three aliquots from each treatment for each sample ID, for fGCM and fTHM (Table S1), and differences among treatments were similarly assessed using mixed effects models (R package: *lme4;*  Bates *et al.,* 2015) with the killer whale ID, with nested sample ID, considered as the random effect. For each hormone, the full model with Treatment as predictor of SD or CV was compared to the null model using the Akaike’s information criterion with correction for small sample size (AICc) and the goodness of fit was assessed with a χ^2^test. When significant, results from the mixed-effect model were analyzed with a post hoc test of EMM for pairwise comparison (R package: *emmeans*: Lenth, 2024). Model assumptions for normality and homoscedasticity were assessed through evaluation of residuals.

To further determine whether sub-samples are representative of the overall faecal event, a Pearson’s correlation analysis was performed on the mean hormone concentration among treatments (R package: *Hmisc*: Harrell Jr, 2025). As the data set contained repeat samples from some individuals, the analysis was conducted on all samples (*n* = 13) as well as a subset with the second sample from three individuals removed (*n* = 10).

## Results

### Faecal glucocorticoid metabolites

Concentrations of faecal glucocorticoid metabolites (fGCM) in the thirteen samples ranged from 48.14 to 434.52 ng/g (mean: 188.47 ng/g ± 73.64 SD; Fig. 3). With the exception of sample 2021-221F, one or more values from pooled and homogenized replicates fell within the range of the sub-samples (Fig. 3). The model with treatment as predictor of fGCM mean concentrations was identified as the better model (AICc = 326.28) when compared to the null model (AICc = 338.23), but these models were statistically indistinguishable (χ^2^ (2) = 5.26, *P* = 0.07; Fig. 4). Mean fGCM, SD, and CV for each treatment and sample are shown in Table S1.

**Figure 5:**
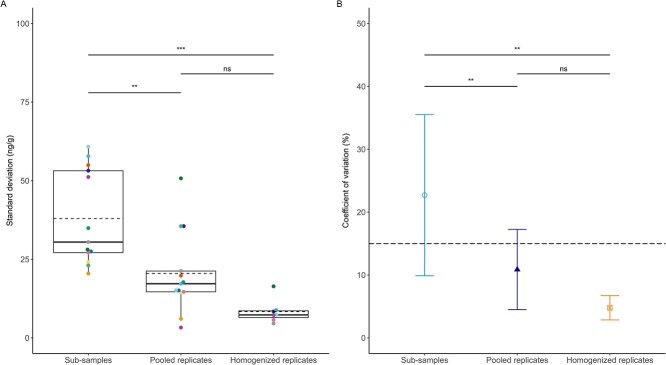
Variation of faecal glucocorticoid metabolites quantified by **A**) SD: ng/g and **B**) CV: % among sub-samples, pooled and homogenized replicates. **A**) The mean SD for sub-samples was significantly higher than mean SD for pooled replicates (EMMeans: *t*(18.5) = 3.5, *P* = 0.007) and for homogenized replicates (EMMeans: *t*(23.0) = 4.86, *P* = < 0.001); no significant difference was found between the SD of pooled and homogenized replicates (EMMeans: *t*(23.0) = 1.99, *P* = 0.14). Boxplots denote median (thick line), mean (dashed line), upper (75%) and lower (25%) quartile (boxes), and largest and smallest value within 1.5 times interquartile range below 25% and above 75% (whiskers). Boxplots are overlaid with each sample/treatment mean, with sample ID indicated by colour. **B**) The mean CV for sub-samples was significantly higher than CV for pooled replicates (10.9 ± 6.4%; EMMeans: *t*(18.5) = 3.52, *P* = 0.006) and for homogenized replicates (EMMeans: *t*(23.0) = 4.12, *P* = 0.001). There was no significant difference between the CVs of pooled and homogenized replicates (EMMeans: *t*(23.0) = 1.24, *P* = 0.44). Bars indicate SD and the horizontal black dashed line at CV = 15% indicates a generally accepted threshold CV for sample replicates.

For SD of fGCM, the full model with treatment as predictor of SD was identified as the better model (AICc = 256.87) and was significantly different from the null model (χ^2^(2) = 21.33, *P* < 0.001; AICc = 282.35). Mean SD of sub-samples (38.0 ± 15.1 ng/g) was significantly higher than SD for pooled replicates (20.5 ± 13.0 ng/g; EMMeans: *t*(18.5) = 3.5, *P* = 0.007) and for homogenized replicates (8.37 ± 3.83 ng/g; EMMeans: *t*(23.0) = 4.86, *P* < 0.001; Fig. 5A). There was no significant difference between the SD of pooled and homogenized replicates (EMMeans: *t*(23.0) = 1.99, *P* = 0.14). Similar to SD, treatment was a significant predictor of CV (χ^2^ (2) = 17.87, *P* < 0.001) and mean CV for sub-samples (25.0 ± 14.7%) was significantly higher than CV for pooled replicates (10.9 ± 6.4%; EMMeans: *t*(18.5) = 3.52, *P* = 0.006) and for homogenized replicates (4.8 ± 1.9%; EMMeans: *t*(23.0) = 4.12, *P* = 0.001). There was no significant difference between the CVs of pooled and homogenized replicates (EMMeans: *t*(23.0) = 1.24, *P* = 0.44). In addition, the mean CV of sub-samples was greater than 15%, while the mean CVs of pooled and homogenized replicates were below 15 and 10%, respectively, which are commonly used thresholds for sample replicates (Fig. 5B). The CV for homogenized replicates was below 15% in all samples (*n* = 7).

### Faecal thyroid hormone metabolites

**Figure 6:**
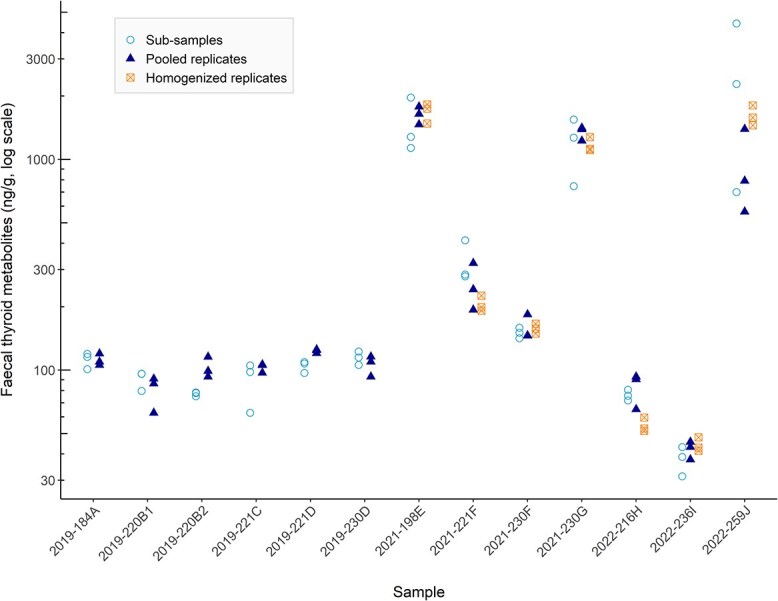
Concentration of faecal thyroid metabolites (ng/g dried faeces) in 13 killer whale (*Orcinus orca*) faecal samples by treatment (sub-samples × 3, pooled sample replicates × 3 and homogenized pooled replicates × 3). Sample names indicate the year of collection followed by the ordinal day and a unique letter identification for each individual (data include a repeat sample from three individuals). Concentrations (*y*-axis) are displayed on log10 scale to better visualize differences across several orders of magnitude.

**Figure 7:**
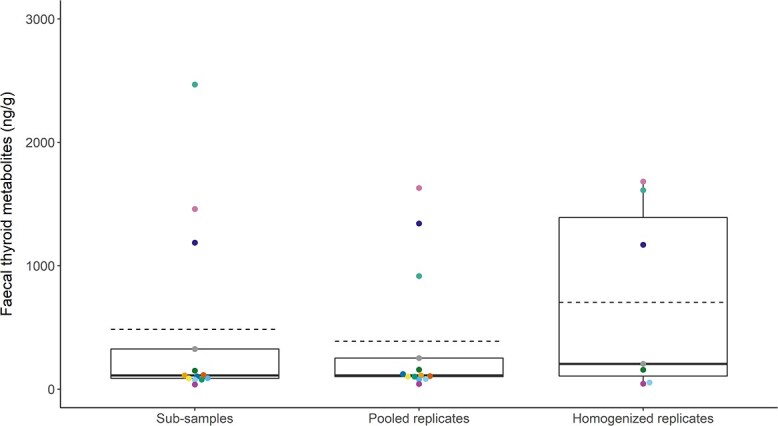
Concentrations of faecal thyroid metabolites (ng/g) by treatment group. Model comparison indicated that treatment group was not a significant explanatory factor for mean concentrations of fTHM (χ^2^(2) = 0.96, *P* = 0.61). Boxplots denote median (thick line), mean (dashed line), upper (75%) and lower (25%) quartile (boxes), and largest and smallest value within 1.5 times interquartile range below 25% and above 75% (whiskers). Boxplots are overlaid with each sample/treatment mean, with sample ID indicated by colour.

Concentrations of faecal thyroid hormone metabolites (fTHM) ranged from 31.31 to 4422.67 ng/g (mean: 493.10 ng/g ± 719.62 SD) in the thirteen samples, with the majority of samples (9 of 13) measuring less than 200 ng/g in all treatment aliquots (Fig. 6). The model with treatment as predictor of fTHM mean concentrations had a slightly lower AICc value than the null model (AICc = 469.43 and 487.35, respectively), but the two models were statistically indistinguishable (χ^2^ (2) = 0.97, *P* = 0.62; Fig. 7). Mean fTHM, SD and CV for each treatment and sample is shown in Table S1.

**Figure 8:**
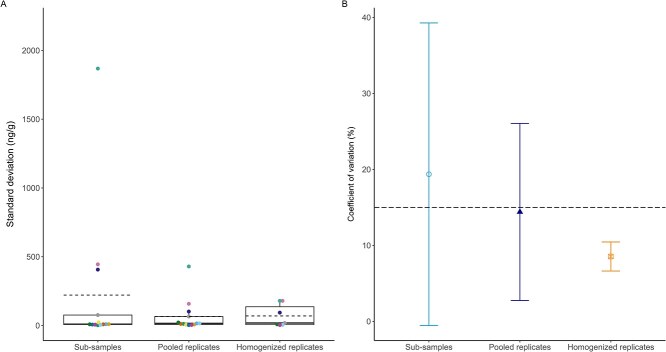
Variation of faecal thyroid hormone metabolites quantified by **A**) SD: ng/g and **B**) CV: % among sub-samples, pooled and homogenized replicates. **A**) Model comparison indicated that treatment group was not a significant explanatory factor for mean SD (χ^2^(2) = 3.00, *P* = 0.22). Boxplots denote median (thick line), mean (dashed line), upper (75%) and lower (25%) quartile (boxes), and largest and smallest value within 1.5 times interquartile range below 25% and above 75% (whiskers). Boxplots are overlaid with each sample/treatment mean, with sample ID indicated by colour. **B**) Model comparison indicated that treatment group was not a significant explanatory factor for mean CV (χ^2^(2) = 5.66, *P* = 0.06). Bars indicate SD and the horizontal black dashed line at CV = 15% indicates a generally accepted threshold CV for sample replicates.

For SD of fTHM, the model with treatment as predictor of SD had a slightly lower AICc value than the null model (AICc = 451.32 and 471.59, respectively), but it was not statistically different from the null model (χ^2^(2) = 3.0, *P* = 0.22). The mean SD for sub-samples (221.0 ± 518.0 ng/g) was greater than for pooled replicates (65.3 ± 119.0 ng/g) and homogenized replicates (69.5 ± 81.3 ng/g), but the differences were not significant (Fig. 8A). Likewise, treatment was not a significant predictor of CV (χ^2^(2) = 5.66, *P* = 0.06); however, similar to fGCM, the mean CV of sub-samples was greater than 15% (19.4 ± 19.9%), while the mean CVs of pooled (14.4 ± 11.7%) and homogenized (8.5 ± 1.9%) replicates were below 15 and 10%, respectively (Fig. 8B). The CV for homogenized replicates was below 15% in all samples (*n* = 7).

Pearson’s correlation analysis indicated that mean fGCM from sub-samples was highly correlated with fGCM from pooled (*r* = 0.93; *P* < 0.001) and homogenized (*r* = 0.89; *P* = 0.008) replicates; fGCM from pooled and homogenized replicates also showed high correlation (*r* = 0.83; *P* = 0.02); similarly, mean fTHM from sub-samples highly correlated with fTHM from pooled (*r* = 0.81; *P* < 0.001) and homogenized (*r* = 0.94; *P* = 0.002) replicates. Mean fTHM was highly correlated between pooled and homogenized replicates (*r* = 0.92; *P* = 0.002; Table 2).

## Discussion

Collection of killer whale faecal samples can be challenging and highly dependent upon weather and sea state, often resulting in small volume. The goal of this study was to determine how reliably a small or partial portion of a resident killer whale faecal sample accurately represents the hormone metabolite concentrations of the entire faecal event. To test this, we examined the variability of glucocorticoid and thyroid metabolites in killer whale faeces collected from the ocean surface, and quantified within-sample variability using SD and CV for three treatments: sub-samples, pooled sub-sample replicates and homogenized pooled replicates. While our findings indicate that pooling and/or homogenizing the sub-samples reduces variability and therefore will result in a better representation of the faecal sample’s mean hormone metabolite value, even small volume sub-samples (≥1 ml) were found to adequately reflect the overall FHM value of the faecal deposit.

The concentrations of fGCM (48–435 ng/g) and fTHM (31–4423 ng/g) observed in this study likely span the expected physiological ranges for killer whales. Although absolute values differ somewhat from previous research in killer whales (Wasser *et al.,* 2017), this is likely due to differences in the immunoassays used to quantify hormone metabolites. Relative values for fGCM followed expected patterns based on sex and reproductive state (Yehle, 2022), and the EIA used to measure fTHM herein yielded a comparable range and distribution of fTHM in grey whales (Lemos *et al.,* 2020). Assessing within-sample variability across a species’ full physiological range is essential, as prior research has shown differences in FHM distribution across low, medium and high ranges (Millspaugh and Washburn, 2003).

While our study is limited in sample size, analysis of faecal samples collected from resident killer whales at sea was necessary to validate the use of small volume samples for FHM analysis. Killer whales are exceedingly broad in their diets across the various ecotypes. Researchers have been largely unsuccessful in collecting samples from other ecotypes, as the faeces rapidly sink and dissipate, likely due to lower density/lipid content than that observed in resident killer whales. Samples from whales in managed care (primarily fed on a diet of herring), while perhaps more readily available, would introduce uncertainties due to significant differences in diet, sampling conditions and faecal sample consistency. As a result, findings from such samples could not necessarily be extrapolated to wild populations of resident killer whales—a common limitation for studies, which use samples from captive animals. As our study was specifically undertaken to evaluate the partitioning of hormones in a resident killer whale faecal sample collected in the field, the use of samples from whales under managed care was not considered to be informative, as the faecal characteristics differ significantly.

Resident killer whale faecal samples vary in density and consistency (e.g. conglomerated vs dispersed particles), and collection is limited to the accessible and floating portion of the defecated material, which may introduce bias or miss hormone metabolite hot spots within the faeces. Similar to previous work on faecal samples from terrestrial mammals (Millspaugh and Washburn, 2003; Parnell *et al.,* 2015), our findings support pooling and homogenizing faecal samples to reduce within-sample variability. For both fGCM and fTHM, average variability expressed as the CV (i.e. ratio of SD to the mean) was less than 15% in the pooled replicates and less than 10% in the homogenized replicates. A CV of 15% is a commonly accepted threshold of variability for endocrine analyses, usually applied as a QA/QC for inter- and intra-assay variation. While both the CVs of fTHM and fGCM sub-sample treatments exceeded 15%, the mean concentrations of fGCM and fTHM were highly correlated among the three treatment groups, supporting the hypothesis that small sub-samples are unlikely to mis-represent the physiological status of the animal.

Variability (expressed as SD) significantly decreased with pooling and even more so with homogenization for fGCM. This may result from mechanical cell lysis (e.g. from beads), through which a more homogeneous number of antigen molecules are exposed to the assay antibody, leading to higher and more consistent recovery of hormone metabolites. Multiple studies have investigated the effect of extraction methodology on the recovery of hormone metabolites (Palme *et al.,* 2013; Wasser *et al.* 2000), and indicated that vortexing rather than boiling of faecal samples yield comparable percentages (Wasser *et al.* 2000). However, the effect of different mechanical homogenization techniques has not been tested.

**Table 2:** Pairwise correlation matrix of fTHM and fGCM concentrations between treatments (*n* = 13 faecal samples)

	Sub-samples	Pooled replicates	Homogenized replicates
**fGCM**—Sub-samples	1.00 (1.00)		
**fGCM**—Pooled replicates	0.93 (0.94)	1.00 (1.00)	
**fGCM**—Homogenized replicates	0.89 (0.89)	0.83 (0.82)	1.00 (1.00)
**fTHM**—Sub-samples	1.00 (1.00)		
**fTHM—**Pooled replicates	0.81 (0.80)	1.00 (1.00)	
**fTHM**—Homogenized replicates	0.94 (0.93)	0.92 (0.92)	1.00 (1.00)

Values in parentheses represent the outcome of a pairwise analysis without including the second sample from three individuals (i.e. *n* = 10 unique individuals). All correlations were statistically significant with *P*-values <0.05.

Within-sample variability in faecal samples from wildlife has been previously evaluated for glucocorticoids (Millspaugh and Washburn, 2003; Parnell *et al.,* 2015), as these metabolites are often measured to assess chronic stress in wildlife (Wasser *et al.,* 2010; Palme, 2019). More recently, there has been increased interest in incorporating thyroid hormones to evaluate nutritional stress (Ayres *et al.,* 2012; Lemos *et al.,* 2021), but there is a lack of knowledge on the distribution of fTHM within faecal samples. This is the first study that investigated within-sample variability in fTHM. Although we did not demonstrate a statistical difference in SD or CV among treatments, our results indicated that pooling and homogenization reduce the variability between replicates and CV among unmixed sub-samples is more likely to exceed commonly accepted thresholds. However, as fTHM concentrations are highly correlated between treatment groups, our data indicate that sub-samples are representative of the overall faecal hormone value.

There are many potential sources of variation in faecal hormone metabolite measurements that are introduced during sample processing and conducting the assay. While some were controlled for in this study, others were not and may have contributed to the observed variability. For example, salt mass from seawater can add to the apparent dried sample mass, leading to a decrease in the apparent hormone concentrations (Hunt *et al.,* 2019). While separation and careful removal of seawater (e.g. after centrifugation) seems to suffice in mitigating this effect, additional steps such as a freshwater rinse may further prevent inflation of the dried sample mass (Lemos *et al.,* 2020). Immunoassay techniques are sensitive to slight differences in sample processing, extraction, evaporation and resuspension steps; as such, some variation between repeated measurements is expected. True within-sample variability (i.e. not variability introduced by methods) may be due to exposure to the environment or the faecal composition itself. Studies have found higher concentrations of FHMs on the outside of faecal deposits, indicating that exposure to temperature and sunlight may influence values (Wasser *et al.,* 1996; Millspaugh and Washburn, 2003). With killer whale faeces, some portions of the faecal deposit may have been in closer contact with seawater, which could interact with polar steroid hormone metabolites. In addition, certain hormone metabolites are lipophilic and may preferentially accumulate in the lipids within a faecal sample. Killer whale faecal samples, when collected, are not uniform in colour and consistency. Many samples had white or light-coloured portions which resembled lipids and did not mix well when vortexed. Further analyses of resident killer whale faeces, in particular the distribution of fats within faecal samples, may provide additional insights into FHM variability within faeces.

This study is the first to investigate within-sample variability of both glucocorticoid and thyroid metabolites in killer whale faeces and highlights some differences in the distribution of fGCM and fTHM, but overall similar conclusions. When possible, studies using small and partial faecal samples for FHM analysis should consider the variability of hormone metabolites and conduct their own assessments. Faecal samples should be well mixed prior to sub-sampling for FHM analysis whenever possible, and our data indicate that small samples (~1 ml) are generally reliable for quantifying glucocorticoid and thyroid metabolites from resident killer whale faeces.

## Supplementary Material

Web_Material_coaf070

## Data Availability

Data will be made available on request.
