# Supplementary material for: Within-sample variability of steroid and thyroid metabolite measurements in faeces of Northeast Pacific resident killer whales (Orcinus orca)
Source: Conserv Physiol. 2025 Oct 9;13(1):coaf070. doi: 10.1093/conphys/coaf070 (PMC12511938; doi:10.1093/conphys/coaf070)
Supplement: Web_Material_coaf070 [file web_material_coaf070.zip › 2025 Yehle SUPPLEMENTAL WITH ALL DATA.pdf]

Supplementary Materials

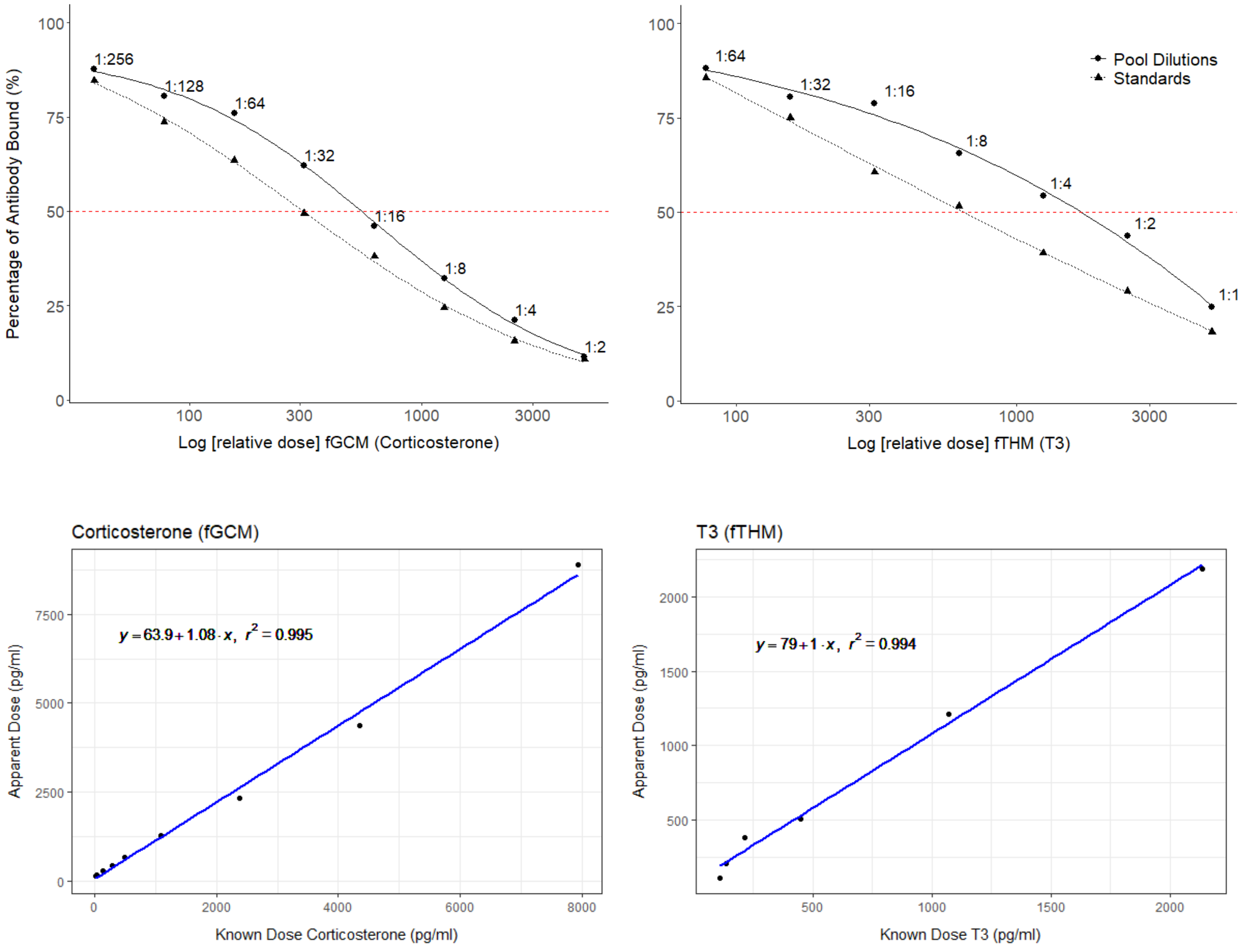

Figure S1. Parallelism (top) and accuracy (bottom) validation results for faecal glucocorticoid (left) and thyroid (right) metabolites (fGCM; fTHM), from Yehle (2022). Parallelism shows serial dilutions of ethanol extracts from a pool of killer whale faeces (circles, solid line) against EIA standard concentrations (triangles, dashed line); parallel displacement over the linear portions of the curves (typically 20-80% antibody binding) indicate that the faecal metabolites bind well to the assay antibody (*F*-test: $F_{1,7} = 1.10$, P = 0.90 for fGCM and $F_{1,6} = 0.87$, P = 0.87, for fTHM). The red dashed line indicates the optimal dilution to achieve 50% binding. Accuracy validation results show an $r^2 > 0.99$ with slopes between 0.9-1.1, indicating the sample matrix does not interfere with antibody binding. Linearity with a slope close to 1.0 indicates that the assay antibody can distinguish between high and low concentrations of the faecal hormone metabolites.

Table S1: Summary of mean concentrations and within-sample variability of glucocorticoid (fGCM) and thyroid (fTHM) metabolites in killer whale faecal samples among three treatments: sub-samples, pooled replicates, and homogenized replicates. Hormone metabolites were extracted and measured in three aliquots for each treatment. For each combination of faecal sample and treatment, the table summarizes mean and standard deviation (SD) of fGCM and fTHM concentrations (ng/g), and the coefficient of variation (CV, %) of the three aliquots.

			fGCM				fTHM			
**Fecal ID**	**Treatment**	**n**	**Aliquots ng/g**	**Mean ng/g**	**SD ng/g**	**CV %**	**Aliquots ng/g**	**Mean ng/g**	**SD ng/g**	**CV %**
2019-184A	Sub-samples	3	395.53<br>434.52<br>425.86	418.64	20.47	4.89	119.43<br>115.58<br>101.07	112.03	9.68	8.64
2019-184A	Pooled replicates	3	389.37<br>397.65<br>385.85	390.96	6.06	1.55	109.51<br>105.73<br>119.82	111.69	7.29	6.53
2019-220B1	Sub-samples	3	204.18<br>101.85<br>106.4	137.48	57.81	42.05	96.13<br>79.62<br>95.95	90.57	9.48	10.47
2019-220B1	Pooled replicates	3	118.71<br>109.09<br>142.56	123.45	17.23	13.96	90.97<br>62.64<br>86.33	79.98	15.2	19
2019-220B2	Sub-samples	3	134.69<br>174.11<br>104.48	137.76	34.92	25.35	78.36<br>77.83<br>75.19	77.13	1.7	2.2
2019-220B2	Pooled replicates	3	163.52<br>197.95<br>173.31	178.26	17.74	9.95	115.56<br>92.97<br>98.94	102.49	11.71	11.42
2019-221C	Sub-samples	3	257.14<br>245.54<br>291.89	264.86	24.12	9.11	98.02<br>105.27<br>62.73	88.67	22.76	25.67
2019-221C	Pooled replicates	3	235.32<br>263.51<br>255.29	251.37	14.5	5.77	106.39<br>105.62<br>97.09	103.03	5.16	5.01

			fGCM				fTHM			
**Fecal ID**	**Treatment**	**n**	**Aliquots ng/g**	**Mean ng/g**	**SD ng/g**	**CV %**	**Aliquots ng/g**	**Mean ng/g**	**SD ng/g**	**CV %**
2021-230F	Sub-samples	3	153.33	181.33	28.11	15.5	158.89	150.28	8.65	5.76
			181.13				141.59			
			209.54				150.35			
2021-230F	Pooled replicates	3	267.94	213.87	50.75	23.73	183.9	158.53	21.98	13.86
			206.41				145.6			
			167.27				146.08			
2021-230F	Homogenized replicates	3	196.25	193.06	16.4	8.5	149.01	157.45	8.53	5.42
			207.63				166.07			
			175.29				157.28			
2021-230G	Sub-samples	3	255.43	196.14	53.19	27.12	747.08	1187.87	406.23	34.2
			180.4				1547.18			
			152.6				1269.35			
2021-230G	Pooled replicates	3	268.44	249.87	35.56	14.23	1225.74	1342.38	101.56	7.57
			272.31				1390.15			
			208.87				1411.24			
2021-230G	Homogenized replicates	3	221.91	231.45	8.42	3.64	1122.27	1169.77	93.5	7.99
			237.84				1277.49			
			234.6				1109.56			
2022-216H	Sub-samples	3	179.83	116.6	60.82	52.16	71.74	76.01	4.48	5.89
			58.51				75.62			
			111.46				80.67			
2022-216H	Pooled replicates	3	191.83	175.58	15.11	8.61	90.39	82.84	15.37	18.55
			172.97				92.98			
			161.95				65.16			
2022-216H	Homogenized replicates	3	174.76	171.04	7.32	4.28	51.43	54.6	4.34	7.94
			175.75				59.54			
			162.6				52.82			

			fGCM				fTHM			
**Fecal ID**	**Treatment**	**n**	**Aliquots ng/g**	**Mean ng/g**	**SD ng/g**	**CV %**	**Aliquots ng/g**	**Mean ng/g**	**SD ng/g**	**CV %**
2022-236I	Sub-samples	3	125.68	106.2	51.18	48.19	38.72	37.71	5.96	15.8
			48.14				43.1			
			144.77				31.31			
2022-236I	Pooled replicates	3	134.21	130.84	3.31	2.53	37.62	42.1	4.07	9.67
			127.59				43.1			
			130.73				45.58			
2022-236I	Homogenized replicates	3	124.48	124.1	7.27	5.86	47.99	44.08	3.48	7.89
			131.18				41.34			
			116.65				42.9			
2022-259J	Sub-samples	3	154.45	175.89	23.06	13.11	700.29	2468.19	1868.19	75.69
			172.93				4422.67			
			200.28				2281.6			
2022-259J	Pooled replicates	3	140.24	167.28	35.55	21.25	564.53	916.81	429.21	46.82
			154.05				791.05			
			207.55				1394.85			
2022-259J	Homogenized replicates	3	203.17	201.26	8.83	4.39	1805.72	1612.65	179.17	11.11
			191.63				1580.49			
			208.98				1451.73			
